# Identifying highly informative genetic markers for quantification of ancestry proportions in crossbred sheep populations: implications for choosing optimum levels of admixture

**DOI:** 10.1186/s12863-017-0526-2

**Published:** 2017-08-24

**Authors:** Tesfaye Getachew, Heather J. Huson, Maria Wurzinger, Jörg Burgstaller, Solomon Gizaw, Aynalem Haile, Barbara Rischkowsky, Gottfried Brem, Solomon Antwi Boison, Gábor Mészáros, Ally Okeyo Mwai, Johann Sölkner

**Affiliations:** 1 Department of Sustainable Agricultural Systems, Division of Livestock Sciences, University of Natural Resources and Life Sciences, Gregor Mendel Straße 33, A-1180 Vienna, Austria; 2 Animal Biodiversity Directorate, Ethiopian Biodiversity Institute, Addis Ababa, Ethiopia; 3 Department of Animal Science, Cornell University, Ithaca, NY USA; 4 Institute of Biotechnology in Animal Production, Department for Agro biotechnology, IFA, Konrad Lorenzstr. 20, 3430 Tulln, Austria; 5 International Livestock Research Institute, Addis Ababa, Ethiopia; 6 International Center for Agricultural Research in the Dry Areas, Addis Ababa, Ethiopia; 7 Institute of Animal Breeding and Genetics, Department of Biomedical Sciences, University of Veterinary Medicine Vienna, Vienna, Austria; 8 Department of Breeding and Genetics, Nofima, Osloveien 1, 1430 Ås, Norway; 9 International Livestock Research Institute, Biotechnology Theme, Nairobi, Kenya

**Keywords:** Ancestry informative markers, Crossbreeding, Ethiopia, Ovine, SNP, Menz, Wollo, Awassi

## Abstract

**Background:**

Understanding the relationship between genetic admixture and performances is crucial for the success of crossbreeding programs. Use of small sets of ancestry informative markers (AIMs) is a cost effective option to estimate the levels of admixture in situations where pedigree recording is difficult. In this study we select AIMs from Ovine 50KSNP data (SNP – single nucleotide polymorphism) and validate their ability in estimating the contributions of parental breeds to get information about optimum admixture levels for smallholders.

**Results:**

We estimated admixture levels using ADMIXTURE software based on 74 AIMs selected from Ovine 50KSNP data. Awassi x Ethiopian fat-tailed crossbred sheep populations kept in government farms and farmer villages in different districts were included in the study. Mean Awassi levels estimated based on the selected AIMs were close to the Awassi levels obtained from pedigree information. Estimates from the 74 SNPs were compared to subsets of 65, 55, 45, 35, 25 and 15 SNPs selected based on both top and bottom fixation index (F_ST_) rank using Spearman’s rank correlation (*r* = 0.862–0.996). A t-test comparison revealed that all SNP subsets had produced similar (*P* > 0.05) estimates with the 74 SNPs, except for the bottom 15 SNPs (*P* < 0.05). However estimates based on 45 and above SNP subsets were relatively close to the fitted regression line (*r* = 0.969–0.996). Association of admixture levels with lamb growth showed that Awassi level affected (*P* < 0.05) eight months weight in both farmer locations, lambs with higher Awassi levels were heavier. Lambing interval of ewes was longer as Awassi level increased, but this drawback was outweighed by the increased productivity of ewes in terms of eight months lamb weight per year.

**Conclusions:**

The results indicate that the Ovine 50KSNP array is a powerful tool to identify small sets of AIMs for admixture studies. The results of this study reveal about 45AIMs to accurately estimate admixture levels. If admixture estimation service with such low cost SNP chip would be available, farmers would have a clear market advantage without the need to rely on incomplete or possibly inaccurate pedigree records. Based on the results presented here, we were able to suggest optimum levels of breed composition for the two farmer environments investigated.

**Electronic supplementary material:**

The online version of this article (doi:10.1186/s12863-017-0526-2) contains supplementary material, which is available to authorized users.

## Background

Small ruminant production is an important agricultural activity and has a substantial contribution to smallholder farmers in generating income and securing food in developing countries [1]. Tropical developing countries rely mostly on non-specialized multipurpose breeds, extensive production systems and breeding is usually uncontrolled. Existing breeds/populations in these countries are well known to adapt to different environmental situations which are often characterized by feed scarcity and disease challenge [2–4]. Contrary to this, breeds in developed world are usually specialized for specific purposes in high input/output systems. Growing demand for food created by rapid human population growth, urbanization and income growth necessitate more productive breeds in developing countries. Local breeds may sometimes be unprofitable as they have limited capacity to respond to improved management because they were not selected for increased production. Crossbreeding is sometimes considered an attractive breed improvement method due to its promise of quick benefit as the result of breed complementarity and heterosis effects [5]. Such combination of multiple breeds could achieve the optimum levels of production, with remarkable results achieved in well-designed selective and crossbreeding schemes [6–9]. The introduction of improved Awassi (Afec-Awassi) to the local Awassi flocks managed by Bedouin farmers in the southern dry region of Israel was successful and made the flocks more profitable [10, 11]. There is growing interest from the government of Ethiopia in sheep crossbreeding to satisfy the rising demand for meat while research efforts on sheep crossbreeding so far did not deliver the anticipated benefit to the smallholder farmers. Mismatching of genotype with environment due to poor feed resource and health management is mentioned as one of the reasons for the little success of crossbreeding [12]. Determining the optimum combination of productivity and adaptability considering the prevailing environment is one of the key issues to be addressed for the success of crossbreeding.

Awassi crossbred rams have been distributed in the highlands of Ethiopia to increase the body size of the indigenous fat-tailed sheep breeds through crossbreeding [13]. However, due to uncontrolled mating, mosaics of crossbreds were produced under farmer’s management. It is very important to investigate how crosses (composites) of different blood levels perform under smallholder management. Setting the level of admixture for different locations based on the existing situation would be an essential part for the success of the on-going crossbreeding program as well as to design larger scale crossbreeding programs in other similar areas. Routine recording of growth of lambs and fertility of female animals has been part of the on-going crossbreeding programs in Negasi-Amba and Chiro villages in Ethiopia. Precise pedigree of the animals is compulsory to identify optimum levels of admixture. However, it has been challenging to keep pedigree records under smallholder farmer’s situation due to uncontrolled mating and poor infrastructure.

Genome sequencing and the subsequent development of single nucleotide polymorphism (SNP) chip data have been extensively used to learn about population stratification and admixture in human populations [14, 15]. Genomic information assists decision making in designing and implementing breed improvement programs although its application in developing countries is limited, mainly due to logistics, lack of knowledge and infrastructure. Even though 50 K and higher density Ovine SNP chips are available with varying level of information for the estimation of admixture levels, genotyping of small subsets of ancestry informative markers (AIMs) might be sufficient to accurately predict the admixture level with a minimal error rate in a cost effective way. AIMs are genetic loci showing alleles with large frequency differences between populations. Thus, the objectives of this study were 1) to identify a small set of AIMs from the Ovine SNP50 BeadChip array to validate the selected AIMs for the estimation of admixture levels in Ethiopian fat-tailed x improved Awassi crossbred populations and 2) to identify the optimal level of Awassi ancestry for different areas based on association of estimated genomic admixture levels with lamb growth and ewe reproductive performance.

## Methods

### The study area and animals

A total of 754 genotypes were obtained from three parental sheep breeds (Menz, Wollo and improved Awassi) and two crossbred populations (Menz x Awassi and Wollo x Awassi) Table 1. Crossbreds here were determined based on phenotypic appearance, available pedigree information and farmers recall on parents. Genotype data for the study were obtained from: 1) sampled sheep kept in three farmers’ villages and two government farms in the highlands of Ethiopia; and 2) the International Sheep Genome Consortium (ISGC, http://www.sheephapmap.org/) database. The three villages were Negasi-Amba, Chiro and Serity. Negasi-Amba and Serity are located in North Shewa whereas Chiro is in the South Wollo administrative zone of the Amhara Regional State in Ethiopia. The two government farms were Debre Berhan Agricultural Research Center (DBARC) and Amed Guya Sheep Breeding and Multiplication Center (AGSBMC). Menz and Wollo sheep breeds are indigenous to Ethiopia, classified as short fat-tailed breeds and they are reared for meat in sub-alpine and cold highland agro ecological zones of Ethiopia [16]. Both breeds are characterized by small size, having coarse wool and very low twinning rate of usually less than 3%. The Awassi sheep breed was introduced from Israel, and is well known for its adaptation to a wide range of environmental conditions and was widely accepted by many Asian and African countries [17, 18]. Crossbreeding of Awassi with Ethiopian fat-tailed sheep has been implemented in the three villages by DBARC since 1997. Details of the breeding program followed by DBARC were described in previous studies [13, 19]. In brief, the project was designed for disseminating high grade exotic crossbred (75% Awassi-25% Menz) rams to farmers for the purpose of upgrading the indigenous Ethiopian fat-tailed sheep breeds (Menz and Wollo) through continuous backcrossing. Indigenous sheep found in Negasi-Amba and Serity are Menz breed whereas the breed in Chiro village is Wollo. During the crossbreeding process, a significant number of admixed populations of Menz x Awassi and Wollo x Awassi have been produced in Negasi-Amba and Chiro villages, respectively whereas the program in Serity was stopped at an early stage.

**Table 1:** Summary of breed, location, sample type and number of observations

Breed/ Population	Village/data source	Sample type	N^a^			
			Ewe	Lamb	UN_ID^h^	Total
Parental breeds						
Imp Awassi^b^	ISGC^e^	-	-	-	23	23
Imp Awassi^b^	AGSBMC^f^	FTA	-	-	18	18
Menz	ISGC^e^	-	-	-	34	34
Menz	Dargegn	Ear tissue	18	-	-	18
Wollo	Chiro	Ear tissue	18	-	-	18
Admixed populations						
MA^c^	Negasi-Amba	Ear tissue	84	158	-	242
MA^c^	Negasi-Amba	Blood using FTA	56	-	-	56
75% MA^c^	Amed-Guya	Blood using FTA	-	-	16	16
WA^d^	Chiro	Ear tissue	149	144	-	293
50% MA^b^	DBARC^g^	Ear tissue	-	-	16	16
MA^b^	Serity	Ear tissue	20	-	-	20
Total			345	302	107	754

^a^N: number of samples

^b^Improved Awassi

^c^MA: Menz x Awassi crossbred population

^d^WA: Wollo x Awassi crossbred

^e^ISGC: International Sheep Genome Consortium

^f^AGSBMC: AmedGuya Sheep Breeding and Multiplication Center

^g^DBARC: DebreBerhan Agricultural Research Center

^h^UN_ID: Sex and class of sheep were not identified

### Sampling, DNA extraction, genotyping and quality control of parental breeds

Ovine SNP50K data from the three parental breeds (*n* = 75 sheep) were used to select AIMs. Ovine SNP50K data (49,034 SNPs) for the two parental breeds, Menz (*n* = 34) and improved Awassi (*n* = 23) are available in ISGC database. Quality control for data obtained from Menz and Awassi sheep was indicated in [20–22]. In brief, the quality control steps included the removal of markers with call rate < 0.99, markers identified during clustering as having atypical X-clustering, SNP with minor allele frequency equal to zero and SNP with discordant genotypes. For the other parental breed, Wollo (*n* = 19), genomic DNA was isolated from ear tissue samples at Holeta Agricultural Research Center, Holeta, Ethiopia. The samples were genotyped using Illumina OvineSNP50 BeadChips (Illumina Inc., San Diego, USA) containing 53,862 evenly spaced SNPs according to the manufacturer protocol. A total of 4494 SNPs and one individual were removed due to SNP call rate of <0.99 and poor genotyping rate (<95% SNP missing rate). After removing SNP with unknown position and merging with common SNPs, a total of 75 individuals from the three breeds (Menz, Wollo and improved Awassi) sharing 47,749 SNPs were available. Data editing and quality check was carried out using PLINK [23].

### Ancestry informative marker selection

SNPs passing the quality control criteria and common to the three breeds (*n* = 47,749) were used to select the AIMs. The two indigenous Ethiopian populations were merged as one breed as they are very close to each other having F_ST_ value of 0.004. A total of 150 SNPs that showed large differentiation between local breeds and exotic Awassi were selected based on their F_ST_ values. The National Center for Biotechnology Information (NCBI) database (http://www.ncbi.nlm.nih.gov/) was used to identify nucleotide sequences in the genomic region for each of selected SNP (100 base pair in both directions). Top F_ST_ ranked 105 SNPs markers were selected from a list of 150 SNPs considering their compatibility in designing primers.

### DNA extraction and genotyping of admixed populations

A total of 643 admixed and 36 pure sheep samples were included in the admixture analysis. Samples from Negasi-Amba (*n* = 298), Chiro (*n* = 293) and Serity (*n* = 20) were obtained from farmers village for DNA extraction. Furthermore, crossbreds with exotic inheritance of 50% Awassi (*n* = 16) and 75% Awassi × 25% Menz (*n* = 16) were collected from DBARC and AGSBMC, respectively. Pure Awassi (*n* = 18) and pure Menz (*n* = 18) samples were collected from AGSBMC and Dargegn village, respectively and included in the admixture analysis as reference. The samples were ear tissues collected with Allflex ear tissue sampler, except for 90 blood samples collected with FTA cards. Ear tissue samples were collected between May and June 2012, whereas the FTA samples were collected in June 2009. Genomic DNA of the ear tissue samples collected from admixed crossbred populations and purebreds was extracted at Holleta Agricultural Research Center. Genome DNA extraction from FTA card was done at the Austrian Technology Institute laboratory, Tulln, Austria.

The AIM SNPs were analyzed using the SequenomMassARRAYiPLEX system (Agena Biosciences, USA) at Veterinary Medicine University laboratory, Tulln, Austria. In short, a section of DNA containing a SNP was amplified from each individual by polymerase chain reaction (PCR), followed by a high-fidelity single-base primer extension reaction over the SNP being assayed, using nucleotides of modified mass. The different alleles therefore produced oligonucleotides with mass differences that could be detected using highly accurate Matrix-Assisted Laser Desorption/Ionization Time-Of-Flight mass spectrometry [24].

Three multiplex assays, each screening for 36, 36 and 33 SNPs, were designed using Assay Design Suite v1.0 software (Agena Biosciences, USA) software. SNP genotyping was performed using the iPLEX® GOLD Complete Genotyping kit with SpectroCHIPs® II in the 384 format (Agena Biosciences, Germany), according to the manufacturer’s protocol, with a single modification: To reduce unspecific primer extension, 5 ng sheared salmon sperm DNA (Invitrogen, Austria) per reaction was added to the PCR mastermix. Results were analyzed with the Sequenom Typer 4.0 software (Agena Biosciences, USA).

### Growth and reproductive performance data

Phenotypic data on growth and reproductive performances of crossbreed sheep having different Awassi levels were collected from flocks in Negasi-Amba and Chiro crossbreeding villages. Data for lamb growth were collected between May and July, 2012. Most of the lambs were at about 8 months of age at sampling time and therefore this weight was considered for the study. This age is also close to the market age of lambs in those areas. Data on ewe lambing interval and number of lambs weaned per ewe per year were obtained from database available at DBARC. Ewes with at least three lambing were considered for this study.

### Body condition score

Sheep body condition score is visual assessment of body fat and muscle on the live sheep. It is independent of body size which can provide an acceptable and useful estimate of the proportion of fat and muscle in live sheep. Data on body condition score were collected from each individual sheep by feeling the muscle and fat cover around the end of short ribs and over the backbone using the balls of the fingers and the thumb. Level of fatness and muscle was scored from 1 (emaciated) to 5 (obese or extremely fat). Full description of sheep body condition scoring is indicated in Additional file 1: Table S1.

### Best and poor performing an van imals

Ewes and lambs with best and poor performance were chosen within each of the two locations to test if there is association between performance and Awassi levels. Ewes were graded based on lambing interval and number of lambs having reached weaning age, whereas lambs were graded based on their eight months weight.

### Estimation of ancestry in admixed population

The ancestry contribution was estimated for the admixed population using 74 top ranked AIMs selected by their F_ST_ value. Admixture level of each animal in the crossbred population was estimated using the software ADMIXTURE v1.2.3 [25] assuming two (*K* = 2) parental populations. This software applies a maximum likelihood-based clustering algorithm that places individuals into two predefined clusters. Both unsupervised (prior population information was ignored) and supervised (with prior population information) computation methods were used. Individual admixture estimates from supervised and unsupervised admixture analyses were compared with Spearman’s correlation coefficients.

### Validation of selected AIMs

Validity of the 74 SNPs in estimating the blood level of Awassi was assessed by comparing the ancestry estimate based on the 74 AIMs with pedigree information. Ancestry contribution was also estimated based on subsets of 65, 55, 45, 35, 25 and 15 SNPs selected based on both top and bottom F_ST_ ranked SNPs in order to assess the possibility of reducing AIMs. Spearman’s rank correlation coefficient was used between each of the subsets and top ranked 74 SNPs. The accuracy and validity of ancestral estimates were also assessed using root mean square error (RMSE) which was used as a summary measure of precision in the estimate of individual ancestry proportion.

$$ RMSE=\sqrt{\frac{1}{n}\sum {\left({\widehat{y}}_i-{y}_i\right)}^2} $$

Where *y*_*i*_ is the observed admixture level for the i^th^ observation and ${\widehat{y}}_i$ is the predicted value and n is the number of pairs of values of observed and predicted. Individual admixture estimate based on 74 SNPs was plotted against each SNP subset. R statistical software (R Development Core Team, 2013) was used for this statistical analysis and visualizing the results in graphs.

### Association of admixture levels with performances

For the analysis of growth and reproductive performance, lambs and ewes were classified into six genetic groups based on the admixture level estimated from the genotype. The groups were 0% Awassi (pure local breeds), 0 to <12.5%, 12.5 to <25%, 25 to <37.5%, 37.5 to <50% and equal or above 50% Awassi levels. Growth, lambing interval, number of lambs weaned per ewe per year were analyzed using PROC MIXED of SAS/STAT (SAS Institute Inc., 2009). Fitting location by genetic group analysis was not appropriate as most of the lambs in Menz location were below 25% Awassi level. Thus, within location analysis was implemented.

The model to analyze lamb 8 month weight for each of Negasi-Amba and Chiro location was:

$$ {y}_{\mathrm{i}\mathrm{jkl}}=\kern0.5em \mu +{b}_{\mathrm{i}}+{s}_j+{f}_k+{\varepsilon}_{ijkl} $$

Where, *y*_*ijkl*_ is lamb eight months weight, *μ* is the overall common constant for all values of y, *b*_*i*_ is the fixed effect of lamb Awassi level, *s*_*j*_ is the fixed effect of sex, *f*_*k*_ is the random effect of owner of lambs and *ɛ*_*ijkl*_ is the residual effect.

The model to analyze lambing interval and number of lambs weaned per ewe per year for each of Negasi-Amba and Chiro location was:

$$ {y}_{ijk}=\kern0.5em \mu +{b}_i+{f}_j+{\varepsilon}_{ijk} $$

Where, *y*_*ijk*_ is lambing interval or number of lambs weaned per ewe per year, *μ* is the overall common constant for all values of y*, b*_*i*_ is the fixed effect of ewe Awassi level, *f*_*j*_ is the random effect of owner of ewes and *ɛ*_*ijk*_ is the residual effect. PROC GLM SAS/STAT [26] was used to analyze the association of ewe and lamb performance levels withAwassi level.

The effect of lamb and ewe performance on Awassi level was further analyzed as:

$$ {y}_{ij}=\kern0.5em \mu +{p}_i+{\varepsilon}_{ij} $$

Where, *y*_*ij*_ is either of the dependent variables in each location (Awassi level for lambs or ewes, 8 months weight and body condition score of lambs, lambing interval or number of lambs weaned per ewe per year)*, μ* is the overall common constant for all values of y, *p*_*i*_ is the fixed effect of ewe or lamb performance level (poor, medium and top) and *ɛ*_*ij*_ is the residual effect.

## Results

### Characteristics of selected markers

A total of 105 SNPs were selected as AIMs from Ovine 50KSNP data based on highly differentiating F_ST_ values between improved Awassi and Ethiopian fat-tailed breeds. The AIMs were distributed across the genome (Additional file 2: Table S2). A quality control in PLINK v1.9 (Purcell et al., 2007) removed SNPs with genotype call rate of <0.9 and individuals with missing rate of <0.9. A total of 74 SNPs were left and used to estimate the individual admixture levels in the crossbred population. Mean, minimum and maximum F_ST_ values of these 74 markers were, 0.81, 0.76, and 0.95, respectively.

### Population and individual ancestry estimates

**Fig. 1:**
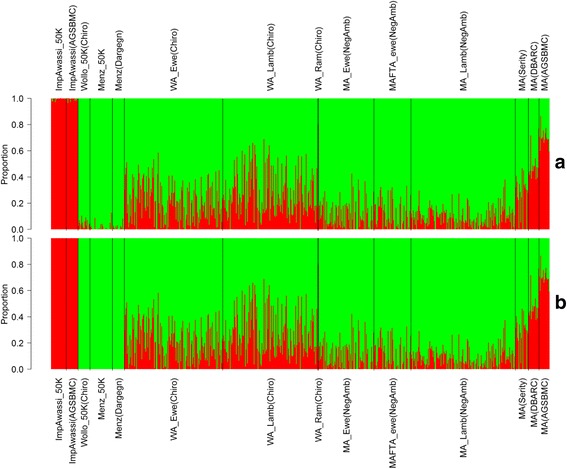
Bar plots of individual ancestry estimates from an unsupervised (**a**) and a supervised (**b**) structure analysis of crossbred populations at K = 2. Individuals were represented by *vertical line* divided in to 2 colors, *red color* indicating the proportion of Awassi and *green color* the proportion of Ethiopian fat-tailed. Each population was separated by black line. The location is indicated in brackets. WA = Wollo x Awassi crossbred, MAFTA = Menz x Awassi crossbred: these samples were collected 3 years before the others using FTA cards, WA = Wollo x Awassi crossbred population, MA = Menz x Awassi crossbred population, DBARC = DebreBerhan Agricultural Research center, AGSBMC = AmedGuya Sheep Breeding and Multiplication Center

Bar plots of individual ancestry estimates for the unsupervised (A) and supervised (B) ancestry analysis from the ADMIXTURE software are presented in Fig. 1. Results were clustered into locations and populations. Significantly different admixture levels were found between locations (Chiro and Negasi-Amba). The average proportion as well as range of Awassi levels was higher in Chiro village compared to the Negasi-Amba village. The current mean ± standard deviation (SD) proportion of Awassi level in Chiro sheep flocks was 21.1 ± 14.71 and 27.5 ± 17.13% for ewes and lambs, respectively. In Negasi-Amba, Awassi levels were 11.0 ± 10.53 and 9.0 ± 7.36% for ewes and lambs, respectively. Proportion of Awassi has been at an increasing trend in Chiro site as evident from the proportion of Awassi level being higher in replacement animals (lambs) than in ewes (Fig. 1). However, in Negasi-Amba the Awassi level looks static or in a decreasing trend. Crossbred ewe population in Negasi-Amba was slightly lower than the crossbred population 3 years ago (Fig. 1).

Notable level of Awassi introgression was observed in pure Ethiopian fat-tailed sheep populations collected from three different locations. The Awassi introgression was higher in Wollo sheep breed (4.0%) than Menz sheep breed collected from two different locations (Negasi-Amba and Dargegn) with a range of 1.2–1.3%. The Spearman’s rank correlation between the individual ancestry fractions estimated by supervised and unsupervised analysis was very high (*r* = 0.990). Fitted linear regression line of the supervised on unsupervised (blue line) for crossbred population perfectly matched the diagonal line (red color) (Additional file 3: Figure. S1). Observations deviated from the diagonal lines at lower and upper end was due to the observed admixture in pure Ethiopian local and improved Awassi breeds, respectively in the unsupervised mode.

### Genome estimated admixture levels and pedigree information

Awassi, levels estimated based on the selected AIMs were close to those obtained from pedigree information for half bred (50% Awassi), 75% Awassi and pure bred (100% Awassi). Especially the estimate was very close for the pure Awassi with mean value of 99.31% with 95% confidence 98.8 to 99.8% (Table 2) and correlation coefficient of 0.98.

**Table 2:** Mean, 95% confidence interval (CI), standard deviation (SD), minimum (Min) and maximum (Max) values of the Awassi level estimated based on 74 SNPs for each category of pedigree admixture level

Awassi level from Pedigree	N^a^	Source			Estimated from 74 SNPs		
			Mean	95% CI	SD	Min	Max
50%	18	DBARC^b^	46.04	43.3-47.7	3.54	40.66	51.86
75%	17	AGSBMC^c^	71.22	69.3-75.0	4.61	60.87	78.56
100%	17	AGSBMC^c^	99.31	98.8-99.8	1.02	97.06	100.00

^a^N: number of observations

^b^DBARC: Debre Berhan Agricultural Research Center

^c^AGSBMC: Amed Guya Sheep Breeding and Multiplication Center

### Estimation of ancestral contributions using different subsets of AIMs

**Fig. 2:**
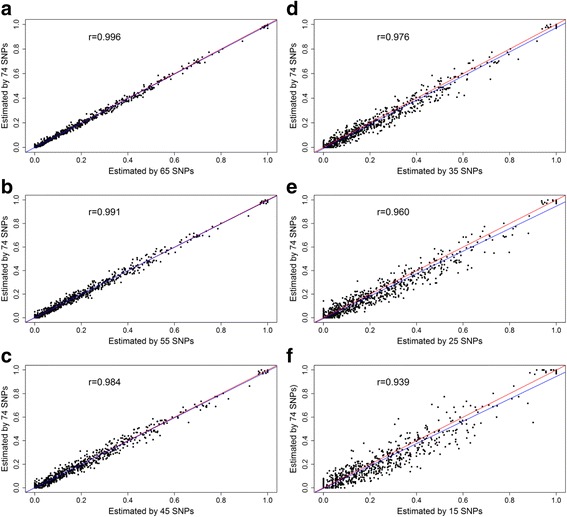
Spearman’s correlation coefficient (r) values and scatter plot of individual Awassi level estimated by top 74 SNPs vs. individual Awassi level estimated by top 65, 55, 45, 35, 25, and 15 SNPs (see panels **a**-**f**, respectively). In each plot the blue line is the linear regression line of individual Awassi level estimated by 74 SNPs on estimated by different subsets, and red line is the diagonal line with perfect linear relationship

**Fig. 3:**
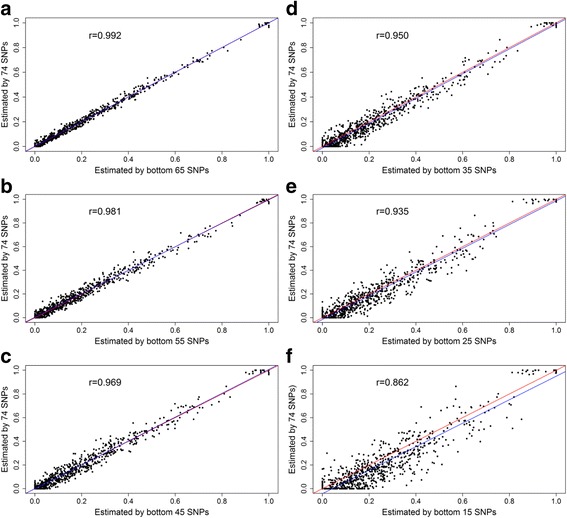
Spearman’s correlation coefficient (r) values and scatter plot of individual Awassi level estimated by top 74 SNPs vs. individual Awassi level estimated by bottom 65, 55, 45, 35, 25, and 15 SNPs (see panels **a**-**f**, respectively). In each plot the blue line is the linear regression line of individual Awassi level estimated by 74 SNPs on estimated by different subsets, and red line is the diagonal line with perfect linear relationship

Spearman’s rank correlations (r) between the Awassi levels estimated using top 74 F_ST_ ranked AIMs and subsets 65, 55, 45, 35, 25 and 15 AIMs selected both from top and bottom F_ST_ ranges are presented in Figs. 2 and 3, respectively. Strong correlation coefficient values were found in the range of 0.862 to 0.996. The r values decreased with decreasing number of AIMs in the subset. Correlation was higher for the top ranked subsets. The difference between correlations based on the top and bottom ranked F_ST_ was higher as the number of SNPs in the subset decreased. Among the SNP subsets the lowest root mean square error (RMSE) was found in the top 65 SNP set (0.013), with an increase to 0.020, 0.028, 0.037, 0.050 and 0.067 with top ranked 55, 45, 35, 25 and 15 AIMs, respectively. Bottom 65, 55, 45, 35, 25 and 15 SNPs resulted in RMSE values of 0.017, 0.028, 0.037, 0.045, 0.058 and 0.089, respectively. A t-test comparison between each subset and the top 74 SNPs (having mean ± SE value of 0.222 ± 0.0092) revealed that all SNP subsets had produced similar (*P* > 0.05) estimates with the mean value estimated based on the 74 SNPs, except for the bottom 15 SNPs (*P* < 0.05) (Table 3). These strong correlations, t-test comparisons and low RMSEs clearly show that all subsets of SNPs, except the one with 15 SNPs, provided reasonable estimates of ancestry compared to the top 74 AIMs. However estimates based on 45 and above SNP subsets was relatively more accurate as the values were relatively close to the fitted regression line (Figs. 2a-c and 3a-c). The estimates were consistent and reproducible for SNPs selected either from top or bottom of the initial set.

**Table 3:** Mean Awassi level estimated by different subset of top ranked and bottom ranked SNPs and t-test results comparing each subset with the reference top 74 selected SNPs

Number of SNPs per AIMs^a^	Number of animals	Selected from top			Selected from bottom		
		Mean ± SE^c^	RMSE	*P*-value^b^	Mean ± SE^c^	RMSE^d^	*P*-value^b^
65	755	0.224 ± 0.0092	0.013	0.891	0.221 ± 0.0092	0.017	0.9121
55	755	0.222 ± 0.0092	0.020	0.984	0.220 ± 0.0092	0.028	0.8576
45	755	0.224 ± 0.0092	0.028	0.906	0.221 ± 0.0090	0.037	0.9413
35	755	0.235 ± 0.0092	0.037	0.314	0.234 ± 0.0090	0.045	0.3557
25	755	0.240 ± 0.0094	0.050	0.222	0.237 ± 0.0089	0.058	0.2635
15	755	0.239 ± 0.0093	0.067	0.207	0.267 ± 0.0087	0.089	0.0004

^a^Each subset was compared with the top 74 SNPs (mean ± SE = 0.222 ± 0.0092)

^b^ *P* = probability value of t-test,^c^SE = standard error, ^d^RMSE = root mean square error

### Association of admixture level with lamb growth and ewe reproductive performances

Regardless of different Awassi level group, both location and lamb sex fitted in the model significantly (*P* < 0.05) affected lamb weight at eight months of age (Additional file 4: Table S3). Lambs in Chiro site were significantly heavier at eight months compared to lambs in Negasi-Amba. Generally, males were heavier than females.

Awassi level significantly affected the eight months weight in both locations whereas the sex effect was significant only in Chiro village (Additional file 4: Table S3). The results indicated that pure local lambs in both locations had similar live weight at eight months. In Negasi-Amba site, pure Menz sheep were lighter (*P* < 0.05) at eight months compared to 0 to <12.5% and 12.5 to <25% Awassi level whereas similar (*P* > 0.05) to highest level of Awassi (25 to <37.5%) in that location. However in Chiro site, lamb eight months weight increased as the Awassi level increased up to 50%. Lambs with Awassi level above 50% were not different (*P* > 0.05) from the previous group (37.5 to <50% Awassi level). Male lambs were heavier (*P* < 0.05) than females in Chiro site.

**Fig. 4:**
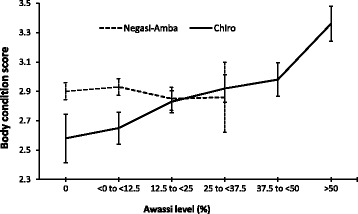
Least square means of body condition score for lambs having different Awassi levels in Negasi-Amba and Chiro site

Body condition scores for the crossbred population by Awassi level in Negasi-Amba and Chiro are presented in Fig. 4. In Negasi-Amba, the body condition score slightly increased for the first crossbred group (0 to <12.5% Awassi) and then declined; whereas in Chiro, the lamb body condition improved significantly with the increasing Awassi level.

Within location analysis for lambing interval, number of lambs weaned per ewe per year and body condition score are presented in Additional file 5: Table S4. In both locations, the effect of Awassi admixture on lambing interval was significant (*P* < 0.05) and the number of lambs weaned per ewe per year and BC score did not vary. Lambing interval was longer as the Awassi level increased in both locations. Number of lambs weaned per ewe per year also showed a non-significant decreasing trend with increasing Awassi level.

### Association of extreme performances of lamb growth and ewe reproduction to Awassi level

Higher within population variations in lamb growth and ewe reproductive performance were observed due to crossbreeding in both locations. The weight of best performing 13% cohort lambs at eight months of age was 5.9 and 10.4 kg higher than the population mean in Negasi-Amba and Chiro, respectively (Additional file 6: Table S5). In both locations best performance of lambs was associated with the level of Awassi. This performance increase was not significant (*P* > 0.05) in Negasi-Amba (6.8 to 10.1% Awassi level), but highly significant (*P* < 0.001) in Chiro village. Here the best performing lambs had an average Awassi level of 37.1%, while the medium and worst performing lambs had 25.2% and 17.7% Awassi level, respectively.

Similarly in ewes, the difference in lambing interval between the best performing and overall mean of ewes in Negasi-Amba and Chiro was 62 and 79 days, respectively. However, reproductive performance was not associated (*P* > 0.05) with Awassi level in any location (Additional file 7: Table S6).

**Fig. 5:**
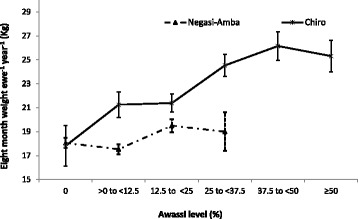
Mean eight months weight produced per ewe per year in Negasi-Amba and Chiro sites

Combined number of lambs weaned per ewe per year and lamb growth might be a useful measure to evaluate the overall productivity of crossbreeding. Eight months lamb weight per ewe per year was calculated by multiplying number of lambs weaned by each ewe in each year of a given Awassi level by assumed post weaning to eight month survival rate of 0.95. Then the value multiplied by eight months weight of the corresponding Awassi level of lamb. The values were calculated considering an important assumption of 95% lamb survival rate between weaning and eight months for all Awassi levels. The eight months lamb weight per ewe per year for each Awassi level group is shown in Fig. 5. The inferiority of ewes in lambing interval due to increased Awassi level was outweighed by the fast growth of lambs. In both locations ewe productivity in terms of eight months lamb weight per year increased with Awassi level. Higher than 50% Awassi levels in Chiro and 25% in Negasi-Amba seem to reach a productivity plateau.

## Discussion

### Selection and validation of AIMs in ancestry estimation

Measuring levels of genomic admixture is of interest in the field of biomedical as well as livestock improvement research. In this study we selected AIMs to infer genome ancestry in the Awassi x Ethiopian fat-tailed crossbred populations and to identify optimum levels of exotic Awassi introgression into Ethiopian fat-tailed sheep under smallholder farmer’s situation. A 1.2 to 4% admixture of Awassi was detected in the pure Ethiopian fat-tailed sheep breeds using unsupervised analyses. These results could possibly be because of the introduction of Awassi sheep at the beginning of 1980’s or, more likely, unsupervised admixture analysis failing to separate the two ancestral breeds due to historic common ancestry. However, supervised vs. unsupervised admixture analysis showed the Awassi introgression had negligible effect on the individual admixture level estimates in crosses (Fig. 1).

We identified a total of 74 SNPs from the Ovine50K SNP data as AIMs. The SNPs were selected based on their F_ST_ values showing highest levels of allele frequency differentiation between the two parental breeds. These AIMs provided close estimation with pedigree information. Correlation coefficient between Awassi level based on pedigree information and admixture estimates from 74 SNP data obtained in this study (*r* = 0.98) was higher compared to the correlation value of 0.96 obtained from ~500 AIMs suggested to predict breed composition in cattle [27]. These results were in agreement with the correlation values in the range of 0.89 to 0.96 reported for different human populations in prediction of admixture level using selected AIMs [28]. Sölkner et al. [29] proposed that individual admixture levels were estimated more accurately based on the genomic data using panels of pure reference animals, compared to estimation based on pedigree. The reason for this difference is the assumption in pedigree based analyses that each offspring inherits half of the genes from each parent and a quarter from each grand-parent, without considering recombination of parental chromosomes in the crossbreeding process [29]. Thus it gives only expected value without considering actual combination of genes. In addition, pedigree recording errors would create additional bias in both under small and large scale farming systems [30]. Use of AIMs for accurate estimation of individual and population admixture levels has also been reported [27, 29, 31, 32]. Pure Awassi samples with full pedigree information were estimated more accurately based on the 74 markers than the 50% and 75% Awassi sheep. The highest precision in the pure Awassi population was expected, as the effect of recombination and recording error is minimal for the pure population.

Ancestry prediction with AIMs also provided reproducible results when the number of SNPs was reduced. This was proved by very strong correlation (0.969 to 0.996) and low RMSE (0.013 to 0.037) observed between the 74 and subsets of 65, 55 and 45 SNPs selected either from top or bottom after ascending based on their F_ST_ values. The RMSEs observed in this study were lower than the reported values of 0.090 and 0.182 when the ancestry estimate based on top 20 and 50 AIMs compared with the true ancestry [15], and comparable with RMSE = 0.026 based on estimate from 105 AIMs and pedigree information in humans [28].

The number of markers required for population assignment depends on the populations under consideration. Our results showed about 45 SNPs selected in any way from the list of 74 SNPs were good enough for accurate estimation of the level of ancestry in the admixed Awassi and Ethiopian fat-tailed populations. The number of markers required to estimate the level of admixture in our study was fewer when compared with ~500 top F_ST_ ranked SNPs required to estimate the admixture level of Swiss Fleckvieh breed [27]. This was attributed to the Ethiopian sheep breed and improved Awassi being genetically more divergent (F_ST_ = 0.76–0.95 for 105 SNPs) compared to the divergence of the ancestral populations of Swiss Fleckvieh (Simmental and Red Holstein Friesian, F_ST_ = 0.623–0.783 for the top 98 SNPs) [15]. Ding et al. [15] suggested as few as top 20 ranked SNPs for accurate classification of ancestral population in humans, where the maximum F_ST_ value was 0.956.

In many developing countries, livestock crossbreeding has been implemented with poor or no pedigree recording. Thus, AIMs would provide a great opportunity to estimate the level of admixture in a cost effective way. Currently, the price or cost per SNP is in the range of about €0.04 to 0.15 for low density panels; highly dependent on the method and number of samples to be genotyped at a time. During data collection many farmers showed interest to pay for the breed composition information. Such information on accurate breed composition, particularly in Chiro site, has been important to sell breeding rams to surrounding farmers as well as other customers coming from other parts of the country. If a very low cost SNP chip would be available for this purpose, farmers would have a clear market advantage without the need to rely on incomplete or possibly inaccurate pedigree records.

### Effect of farmers and location on best levels of Awassi admixture

Effects of location and farmers clearly influenced sheep performances and the level of Awassi admixture in two Ethiopian sheep populations (Additional file 8: Fig. S2**)**. Factors associated to variation in sheep performances and admixture proportions among location and farmers need to be investigated; however it might be associated with sheep flock size, environment, resource of farmers to keep bigger crossbred animals, level of farmers participation, interest of farmers, input access, variation in knowledge among farmers [33–35]. About 27% of farmers in Chiro had Awassi levels of above 30% in their flocks. Such farmers would have better potential to be upgraded towards breeding ram multiplier with support from the government. This would share the load of government farms engaged in multiplication and dissemination of crossbred rams. This would also be cost effective and more efficient than government farms, as the government farms frequently suffer from budget constraints, lack of facilities and higher risk of disease outbreaks associated with confinement. Ahuya et al. [36] reported the previous government approach based on multiplication and dissemination of exotic bucks failed to bring anticipated change in Kenyan goat crossbreeding. Subsequently, the non-governmental organizations, German Development Cooperation (GIZ) and FARM-Africa initiated a community approach led by farmers and became more successful, significantly improving the livelihoods of resource poor families in Kenya [37].

### Lamb growth and ewe reproductive performances

The identification of the optimum level of exotic genotype considering adaptability of the local and productivity of the exotic breeds will be a crucial step in designing and implementing crossbreeding programs in developing countries. This study revealed that crossbreeding with Awassi has improved body weight of local sheep. The overall lamb eight months weight of 16.8 in Negasi-Ambaand 20.7 kg in Chiro was higher even when compared with the previously reported yearling weights in local sheep breeds of Ethiopia, ranging from 15.7 to 17.4 kg [12, 38–40].

Environmental factors and management highly influenced the performance of crossbreds. Results in this study clearly showed the existence of interaction between Awassi level and location. Pure local lambs in both locations had similar live weight at eight months, while the effect of crossbreeding in terms of lamb weight and body condition score at eight months was more pronounced in Chiro village. The eight months weight of 37.5 to <50% Awassi sheep in Chiro (24.8 kg) was superior to the 75% Awassi reported (23.5 kg) under on-station management at yearling age [12]. It was also higher than the two biggest indigenous breeds of Ethiopia reported 21 kg at 9 months for Washera [41, 42] and 17.8 kg at 8 months for Horro [43] breeds. However, eight months lamb weight and body condition score were not improved beyond 12.5% Awassi in Negasi-Amba village. More pronounced improvement of lamb growth and body condition in Chiro due to the increase in Awassi level might be associated with the farmers improved management to cope with the requirements of higher Awassi levels. Attractive price and increasing demand for higher Awassi level for breeding purpose particularly in Chiro site [13, 44] might have inspired farmers to provide better management for their crossbred lambs. Similar to this study, increased live weight with increasing level of exotic gene was reported [3, 45–47]. Effect of location and interaction of breed by location on live weight of crossbred were also reported in a Kenyan Dorper with Red Maasai crossbreeding study [48]. Better lamb growth and inferior to comparable reproductive performance of Awassi crossbreds compared to local breeds found in this study was in agreement with previous Awassi crossbreeding studies in Ethiopia [13, 19, 49, 50]. The inferiority of ewes in lambing interval due to increased Awassi level however was more than compensated by the fast growth and better survival of lambs as indicated by the superior eight month weight performance per ewe per year (Fig. 5). In both locations productivity of ewe in terms of eight months lamb weight per year increased with Awassi level. Variable plateau for this increase was reached at different admixture levels in the two locations, 37 to <50% in Chiro and 12.5 to <25% in Negasi-Amba (Fig. 5). The number of observations above 25% Awassi in Negasi-Amba was too small for reliable conclusions, however noticeable difference was observed between the two locations at similar Awassi levels. Even no improvement on body condition score (Fig. 4) and lamb eight month weight (Additional file 4: Table S3) was observed beyond 12.5% Awassi level in Negasi-Amba. Increase of lamb weaning weights per ewe with increasing level of exotic genes up to 50% level was also reported [50]. Lambing intervals obtained for the ewes with higher Awassi level (334 days) could fit well in an annual mating strategy aiming to adjust lambing to better seasons which is suggested to improve the profitability of the farm by reducing higher lamb mortality occurringon lambs born during the dry seasons (December to May) [51] .

We strongly suggest that choosing the level of exotic ancestry should consider the existing environment and its potential for improved management. Burrow [6] suggested 25 to 75% adapted genes are required for optimal production depending on the severity of the environment and the level of stress challenge, only exceptionally stressful environments require 100% adaptive genes. Based on our results, up to 50%Awassi level would be suggested for Chiro village and similar areas. In Negasi-Amba however, the current situation could only support lower levels of Awassi admixture. The Awassi level suggested for Chiro site in this study was slightly higher than the recommended 37.5% Awassi level under farmer’s management [52]. A smaller number of lambs with higher Awassi level in the previous study [52] and management change adopted through time might be reasons for this difference. The higher genetic variability of lamb growth in crossbred population particularly in Chiro village (mean = 20.4 kg, coefficient of variation = 28.4%, range = 11.2–36.4 kg) (Additional file 4: Table S3) could be considered as an immense potential for improvement of productivity. Genetic improvement applying efficient selection within the crossbred population and improvement in management should be considered together, which progressively leads to the development of a composite population. Imposing selection on the parental breeds in the sheep breeding and multiplication centers based on the information from the pure lines and crossbreds should also be considered to maximize potential genetic gain [53].

## Conclusions

The OvineSNP50 BeadChip array is a powerful tool to identify small subset of highly informative markers for inferring ancestry. We have established a list of 74 SNPs (ancestry informative markers, AIMs) with high levels of allele frequency differences between the Awassi and Ethiopian fat-tailed sheep. A subset of 45 SNPs selected from this list sufficed to accurately estimate admixture levels with a minimal error rate. This small set of SNPs might be cost effective for smallholder farmers to obtain the crossbreeding levels for their animals. Estimation of admixture levels could be easily integrated in the current Awassi crossbreeding schemes to increase efficiency of breeding programs. Moreover, the use of AIMs would inspire livestock breeding programs by availing breed composition, as opposed to pedigree based estimates of admixture levels, which persists as marked constraint in many developing countries. Genetic improvement applying efficient selection within the crossbred population and improvement in management should be considered together, leading to the development of a potentially robust and adapted composite population, preserving allelic combinations underlying productivity and particular adaptations. While this option should be explored in some areas of Ethiopia, we do not consider crossbreeding of local with exotic breeds the most important route for sheep improvement. Within breed selection of local breeds, applying conventional tools of performance recording and ranking of best animals needs to be widely established and supported.

## Additional files

Additional file 1: Table S1. Descriptions of subjective sheep body condition scoring. (DOC 154 kb)

Additional file 2: Table S2. Selected ancestry informative markers. The marker name, chromosomal position, approximate location of the markers on the chromosome in kilo bases (Kb), two alleles, allele frequency of the first allele for each ancestral population, pairwise F_ST_ values and non-missing allele counts for each population are shown. (DOC 169 kb)

Additional file 3: Figure S1. Scatter plots of individual admixture levels estimated with supervised vs. unsupervised analyses. Blue line represents fitted linear regression line of supervised on unsupervised and red color represents the diagonal line when x = y. (PDF 86 kb)

Additional file 4: Table S3. Least square means ± standard errors of lamb eight months weight for crossbred population by location, different Awassi level groups and sex in Negasi-Amba and Chiro villages. (DOC 48 kb)

Additional file 5: Table S4. Least square means ± standard errors of lambing interval, number of lambs weaned per ewe per year and body condition score for the effect of Awassi level groups and sex in each site. (DOC 45 kb)

Additional file 6: Table S5. Least square means ± standard errors of eight months weight, body condition score (BC) and Awassi level for top ranked and poor performing lambs. (DOC 37 kb)

Additional file 7: Table S6. Least square means ± standand error of Awassi level and reproductive performance for top, medium and worst performing ewes in Negasi-Amba and Chiro sites. (DOC 37 kb)

Additional file 8: Figure S2. Mean Awassi level and total number of crossbred sheep produced by farmers in Negasi-Amba (A) and Chiro (B) villages. Bar plots are indicated the total number of crossbred sheep by an owner and line plots showed the average level of Awassi (%). (PDF 234 kb)

## Abbreviations

AGSBMC: Amed Guya Sheep Breeding and Multiplication Center
AIM(s): ancestry informative marker(s)
DBARC: Debre Berhan Agricultural Research Center
F_ST_: a commonly used measure of population differentiation due to genetic structure
r: Spearman’s rank correlation coefficient
RSME: root mean squared error
SD: standard deviation
SNP(s): single nucleotide polymorphism(s)
