# Supplementary material for: Identifying highly informative genetic markers for quantification of ancestry proportions in crossbred sheep populations: implications for choosing optimum levels of admixture
Source: BMC Genet. 2017 Aug 24;18:80. doi: 10.1186/s12863-017-0526-2 (PMC5571632; doi:10.1186/s12863-017-0526-2)
Supplement: Supplementary file 1 — Descriptions of subjective sheep body condition scoring. (DOC 154 kb) [file 12863_2017_526_MOESM1_ESM.doc]

**Subjective sheep body condition scoring method using spinous and transverse process**

| **Figure** | **Explanation** |
| --- | --- |
| 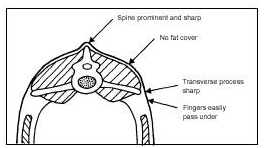 | **Condition 1 (Emaciated)**  Spinous processes are sharp and prominent. Loin eye muscle is shallow with no fat cover. Transverse processes are sharp; one can pass fingers under ends. It is possible to feel between each process. |
| 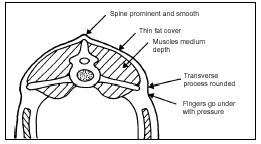 | **Condition 2 (Thin)**  Spinous processes are sharp and prominent. Loin eye muscle has little fat cover but is full. Transverse processes are smooth and slightly rounded. It is possible to pass fingers under the ends of the transverse processes with a little pressure. |
| 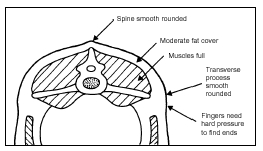 | **Condition 3 (Average)**  Spinous processes are smooth and rounded and one can feel individual processes only with pressure. Transverse processes are smooth and well covered, and firm pressure is needed to feel over the ends. Loin eye muscle is full with some fat cover. |
| 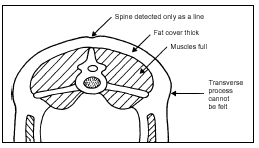 | **Condition 4 (Fat)**  Spinous processes can be detected only with pressure as a hard line. Transverse processes cannot be felt. Loin eye muscle is full with a thick fat |
| 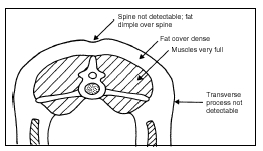 | **Condition 5 (Obese)**  Spinous processes cannot be detected. There is a depression between fat where spine would normally be felt. Transverse processes cannot be detected. Loin eye muscle is very full with a very thick fat |

**Source:** (Thompson and Meyer, 1994)
