# Supplementary material for: Identifying highly informative genetic markers for quantification of ancestry proportions in crossbred sheep populations: implications for choosing optimum levels of admixture
Source: BMC Genet. 2017 Aug 24;18:80. doi: 10.1186/s12863-017-0526-2 (PMC5571632; doi:10.1186/s12863-017-0526-2)
Supplement: Supplementary file 2 — Selected ancestry informative markers. The marker name, chromosomal position, approximate location of the markers on the chromosome in kilo bases (Kb), two alleles, allele frequency of the first allele for each ancestral population, pairwise FST values and non-missing allele counts for each population are shown. (DOC 169 kb) [file 12863_2017_526_MOESM2_ESM.doc]

**Selected ancestry informative markers.** The marker name, chromosomal position, approximate location of the markers on the chromosome in kilobases (Kb), two alleles, allele frequency of the first allele for each ancestral population, pairwise F_ST_ values and non-missing allele counts for each population are shown.

|  | **SNP** | **Chr** | **Position (Kb)** | **A1** | **A2** | **Allele frequency of A1** | | **F_ST_** | **N** | |
| --- | --- | --- | --- | --- | --- | --- | --- | --- | --- | --- |
|  |  |  |  |  |  | **IAW** | **Eth** |  | **IAW** | **Eth** |
| 1 | OAR1_19651513.1 | 1 | 19652 | C | A | 0.00 | 0.84 | 0.81 | 46 | 104 |
| 2 | OAR1_19750493.1 | 1 | 19750 | A | G | 0.15 | 0.92 | 0.76 | 46 | 104 |
| 3 | OAR1_19817567.1 | 1 | 19818 | G | A | 0.02 | 0.91 | 0.88 | 46 | 104 |
| 4 | s40404.1 | 1 | 34229 | A | G | 0.00 | 0.82 | 0.78 | 46 | 104 |
| 5 | OAR1_119469795.1 | 1 | 119470 | G | A | 0.14 | 0.97 | 0.83 | 42 | 104 |
| 6 | s37703.1 | 1 | 146952 | C | A | 0.16 | 0.97 | 0.83 | 44 | 104 |
| 7 | OAR1_211426054.1 | 1 | 211426 | G | A | 0.09 | 0.90 | 0.80 | 46 | 104 |
| 8 | OAR1_215302002.1 | 1 | 215302 | A | G | 0.24 | 0.99 | 0.78 | 46 | 104 |
| 9 | OAR1_215364467.1 | 1 | 215364 | G | A | 0.24 | 1.00 | 0.80 | 46 | 104 |
| 10 | OAR2_15631497.1 | 2 | 15631 | A | G | 0.11 | 0.94 | 0.83 | 38 | 104 |
| 11 | OAR2_28358746.1 | 2 | 28359 | A | G | 0.04 | 0.99 | 0.95 | 46 | 104 |
| 12 | OAR2_53589838.1 | 2 | 53590 | A | G | 0.00 | 0.85 | 0.82 | 46 | 104 |
| 13 | s66123.1 | 2 | 53716 | G | A | 0.02 | 0.83 | 0.78 | 46 | 104 |
| 14 | OAR2_146230561.1 | 2 | 146231 | G | A | 0.02 | 0.83 | 0.76 | 46 | 104 |
| 15 | OAR2_154444578.1 | 2 | 154445 | G | A | 0.17 | 0.97 | 0.81 | 46 | 104 |
| 16 | OAR2_154465026.1 | 2 | 154465 | A | C | 0.20 | 0.98 | 0.80 | 46 | 104 |
| 17 | OAR2_167748075.1 | 2 | 167748 | G | A | 0.17 | 1.00 | 0.86 | 46 | 104 |
| 18 | OAR2_219083907.1 | 2 | 219084 | A | G | 0.13 | 0.91 | 0.76 | 46 | 104 |
| 19 | OAR2_235562127.1 | 2 | 235562 | A | G | 0.07 | 0.89 | 0.81 | 46 | 104 |
| 20 | s39898.1 | 3 | 14786 | G | A | 0.15 | 0.94 | 0.78 | 46 | 104 |
| 21 | s54627.1 | 3 | 25815 | A | G | 0.00 | 0.83 | 0.79 | 46 | 104 |
| 22 | OAR3_180522818.1 | 3 | 180523 | A | G | 0.09 | 0.90 | 0.79 | 46 | 104 |
| 23 | OAR4_37110130.1 | 4 | 37110 | A | G | 0.13 | 0.97 | 0.84 | 46 | 104 |
| 24 | OAR4_56106365.1 | 4 | 56106 | A | G | 0.20 | 0.99 | 0.82 | 46 | 104 |
| 25 | s63232.1 | 5 | 16552 | C | A | 0.00 | 0.79 | 0.76 | 46 | 104 |
| 26 | s04774.1 | 5 | 48865 | A | G | 0.07 | 0.89 | 0.81 | 46 | 104 |
| 27 | OAR5_56291790.1 | 5 | 56292 | A | G | 0.00 | 0.84 | 0.80 | 46 | 104 |
| 28 | OAR6_29577816.1 | 6 | 29578 | A | G | 0.15 | 0.95 | 0.80 | 46 | 104 |
| 29 | OAR6_65652422.1 | 6 | 65652 | G | A | 0.24 | 0.99 | 0.78 | 46 | 104 |
| 30 | OAR6_84531567.1 | 6 | 84532 | G | A | 0.20 | 0.96 | 0.77 | 46 | 104 |
| 31 | OAR6_97185219.1 | 6 | 97185 | G | A | 0.00 | 0.83 | 0.79 | 46 | 104 |
| 32 | OAR6_99879359.1 | 6 | 99879 | A | T | 0.07 | 0.90 | 0.82 | 46 | 104 |
| 33 | s01880.1 | 7 | 30761 | G | A | 0.20 | 0.97 | 0.78 | 46 | 104 |
| 34 | OAR7_89553402.1 | 7 | 89553 | A | G | 0.04 | 0.85 | 0.76 | 46 | 104 |
| 35 | OAR9_20849838.1 | 9 | 20850 | A | G | 0.13 | 0.91 | 0.76 | 46 | 104 |
| 36 | s61067.1 | 9 | 37903 | A | G | 0.13 | 0.91 | 0.76 | 46 | 104 |
| 37 | OAR9_57111594.1 | 9 | 57112 | G | A | 0.15 | 1.00 | 0.87 | 46 | 104 |
| 38 | OAR9_61775166.1 | 9 | 61775 | A | G | 0.09 | 0.96 | 0.87 | 46 | 104 |
| 39 | OAR10_70905390.1 | 10 | 70905 | A | C | 0.17 | 0.94 | 0.76 | 46 | 104 |
| 40 | OAR10_91533055.1 | 10 | 91533 | G | A | 0.07 | 0.87 | 0.76 | 46 | 104 |
| 41 | OAR11_31531641.1 | 11 | 31532 | A | G | 0.22 | 0.99 | 0.80 | 46 | 104 |
| 42 | s64521.1 | 11 | 58802 | G | A | 0.00 | 0.84 | 0.80 | 46 | 104 |
| 43 | OAR11_62400735.1 | 11 | 62401 | G | A | 0.17 | 1.00 | 0.86 | 46 | 104 |
| 44 | OAR12_76153608.1 | 12 | 76154 | G | G | 0.00 | 0.79 | 0.76 | 46 | 104 |
| 45 | s48052.1 | 13 | 3579 | C | A | 0.00 | 0.81 | 0.77 | 46 | 104 |
| 46 | OAR13_8946629.1 | 13 | 8947 | G | A | 0.11 | 0.89 | 0.76 | 46 | 104 |
| 47 | OAR13_73857254.1 | 13 | 73857 | G | A | 0.17 | 0.94 | 0.76 | 46 | 104 |
| 48 | s04113.1 | 13 | 80524 | C | A | 0.02 | 0.85 | 0.79 | 46 | 104 |
| 49 | OAR13_81410207.1 | 13 | 81410 | G | A | 0.00 | 0.85 | 0.81 | 46 | 104 |
| 50 | OAR14_1314896_X.1 | 14 | 1315 | G | A | 0.13 | 0.93 | 0.79 | 46 | 104 |
| 51 | s24630.1 | 14 | 2723 | A | G | 0.02 | 0.92 | 0.88 | 46 | 104 |
| 52 | s03339.1 | 14 | 7903 | A | G | 0.00 | 0.87 | 0.84 | 46 | 104 |
| 53 | s37862.1 | 16 | 1386 | G | A | 0.09 | 0.90 | 0.80 | 46 | 104 |
| 54 | s13627.1 | 16 | 29101 | A | G | 0.00 | 0.86 | 0.82 | 46 | 104 |
| 55 | OAR16_32866225.1 | 16 | 32866 | A | G | 0.13 | 0.93 | 0.79 | 46 | 104 |
| 56 | OAR16_49974943.1 | 16 | 49975 | G | A | 0.07 | 0.88 | 0.79 | 46 | 104 |
| 57 | s33640.1 | 16 | 67333 | G | A | 0.00 | 0.86 | 0.83 | 46 | 104 |
| 58 | s08479.1 | 17 | 58921 | A | G | 0.00 | 0.93 | 0.92 | 46 | 104 |
| 59 | OAR17_64750846.1 | 17 | 64751 | A | G | 0.00 | 0.85 | 0.82 | 46 | 104 |
| 60 | s25024.1 | 17 | 66592 | G | A | 0.05 | 0.97 | 0.93 | 44 | 104 |
| 61 | OAR18_62319441.1 | 18 | 62319 | A | G | 0.15 | 0.97 | 0.83 | 46 | 104 |
| 62 | OAR20_7267196.1 | 20 | 7267 | A | C | 0.00 | 0.92 | 0.91 | 46 | 104 |
| 63 | OAR21_6579878.1 | 21 | 6580 | A | G | 0.15 | 0.98 | 0.84 | 46 | 104 |
| 64 | s52246.1 | 21 | 33311 | A | G | 0.15 | 0.93 | 0.77 | 46 | 104 |
| 65 | OAR21_45614480.1 | 21 | 45614 | A | G | 0.16 | 0.96 | 0.80 | 44 | 104 |
| 66 | OAR22_26079325.1 | 22 | 26079 | C | A | 0.04 | 0.90 | 0.84 | 46 | 104 |
| 67 | OAR23_5044297.1 | 23 | 5044 | G | A | 0.00 | 0.86 | 0.83 | 46 | 104 |
| 68 | s46432.1 | 23 | 52347 | A | C | 0.07 | 0.90 | 0.81 | 46 | 104 |
| 69 | OAR24_8950654.1 | 24 | 8951 | G | A | 0.15 | 0.93 | 0.77 | 46 | 104 |
| 70 | s29758.1 | 25 | 2868 | C | A | 0.02 | 0.91 | 0.87 | 46 | 104 |
| 71 | OAR25_19228950.1 | 25 | 19229 | A | G | 0.15 | 0.94 | 0.78 | 46 | 104 |
| 72 | s10741.1 | 25 | 44633 | G | A | 0.26 | 0.99 | 0.76 | 46 | 104 |
| 73 | OAR26_27745939.1 | 26 | 27746 | G | A | 0.24 | 0.99 | 0.78 | 46 | 104 |
| 74 | OAR26_33341119.1 | 26 | 33341 | G | A | 0.09 | 0.91 | 0.81 | 46 | 104 |
| 75 | OAR1_78868061.1 | 1 | 78868 | G | A | 0.04 | 0.92 | 0.86 | 46 | 104 |
| 76 | OAR1_204671262.1 | 1 | 204671 | A | G | 0.07 | 0.95 | 0.88 | 46 | 104 |
| 77 | OAR1_204686791.1 | 1 | 204687 | A | G | 0.07 | 0.98 | 0.92 | 46 | 104 |
| 78 | OAR2_39209186.1 | 2 | 39209 | A | G | 0.07 | 0.88 | 0.77 | 46 | 104 |
| 79 | OAR2_141221105.1 | 2 | 141221 | A | G | 0.11 | 0.98 | 0.88 | 46 | 104 |
| 80 | OAR2_146458539.1 | 2 | 146459 | G | A | 0.09 | 0.88 | 0.76 | 46 | 104 |
| 81 | OAR3_66722206.1 | 3 | 66722 | A | G | 0.00 | 0.79 | 0.75 | 46 | 104 |
| 82 | OAR3_142190582.1 | 3 | 142191 | C | A | 0.02 | 0.85 | 0.79 | 46 | 104 |
| 83 | OAR3_183826897.1 | 3 | 183827 | G | A | 0.02 | 0.83 | 0.77 | 46 | 104 |
| 84 | OAR4_53537262.1 | 4 | 53537 | C | A | 0.02 | 0.82 | 0.76 | 46 | 104 |
| 85 | OAR5_113136843.1 | 5 | 113137 | G | A | 0.00 | 0.84 | 0.80 | 46 | 104 |
| 86 | OAR6_19690807.1 | 6 | 19691 | G | A | 0.11 | 0.92 | 0.80 | 46 | 104 |
| 87 | OAR7_83073383.1 | 7 | 83073 | G | A | 0.04 | 0.88 | 0.80 | 46 | 104 |
| 88 | OAR7_95938778.1 | 7 | 95939 | A | G | 0.09 | 0.93 | 0.83 | 46 | 104 |
| 89 | OAR9_56685157.1 | 9 | 56685 | G | A | 0.15 | 0.95 | 0.80 | 46 | 104 |
| 90 | s42079.1 | 9 | 58507 | A | G | 0.15 | 0.96 | 0.81 | 46 | 104 |
| 91 | s31237.1 | 9 | 78129 | A | G | 0.09 | 0.96 | 0.87 | 46 | 104 |
| 92 | OAR10_64481741.1 | 10 | 64482 | A | G | 0.15 | 0.97 | 0.83 | 46 | 104 |
| 93 | s70144.1 | 11 | 8373 | G | A | 0.26 | 0.99 | 0.76 | 46 | 104 |
| 94 | OAR11_48144420.1 | 11 | 48144 | G | A | 0.15 | 0.92 | 0.76 | 46 | 104 |
| 95 | s75385.1 | 11 | 61193 | A | G | 0.24 | 1.00 | 0.80 | 46 | 104 |
| 96 | s67736.1 | 12 | 31541 | A | G | 0.09 | 0.90 | 0.79 | 46 | 104 |
| 97 | OAR12_83564723.1 | 12 | 83565 | G | A | 0.13 | 0.93 | 0.80 | 46 | 104 |
| 98 | s69860.1 | 14 | 960 | A | G | 0.02 | 0.91 | 0.87 | 46 | 104 |
| 99 | OAR14_4424829.1 | 14 | 4425 | A | G | 0.02 | 0.85 | 0.79 | 46 | 104 |
| 100 | OAR14_50148641.1 | 14 | 50149 | G | A | 0.09 | 0.90 | 0.79 | 46 | 104 |
| 101 | s05017.1 | 15 | 90028 | A | G | 0.00 | 0.94 | 0.93 | 46 | 104 |
| 102 | OAR17_9906615.1 | 17 | 9907 | G | A | 0.11 | 0.94 | 0.82 | 46 | 104 |
| 103 | OAR17_64771249.1 | 17 | 64771 | A | C | 0.00 | 0.85 | 0.82 | 46 | 104 |
| 104 | OAR20_51262941.1 | 20 | 51263 | G | A | 0.09 | 0.88 | 0.76 | 46 | 104 |
| 105 | DU481531_204.1 | 26 | 6467 | A | G | 0.04 | 0.97 | 0.92 | 46 | 104 |

Chr = chromosome, N = number of observations, IAW = improved Awassi, Eth = Ethiopian fat-tailed breed.
