# Supplementary figures and images for: Identifying highly informative genetic markers for quantification of ancestry proportions in crossbred sheep populations: implications for choosing optimum levels of admixture
Source: BMC Genet. 2017 Aug 24;18:80. doi: 10.1186/s12863-017-0526-2 (PMC5571632; doi:10.1186/s12863-017-0526-2)

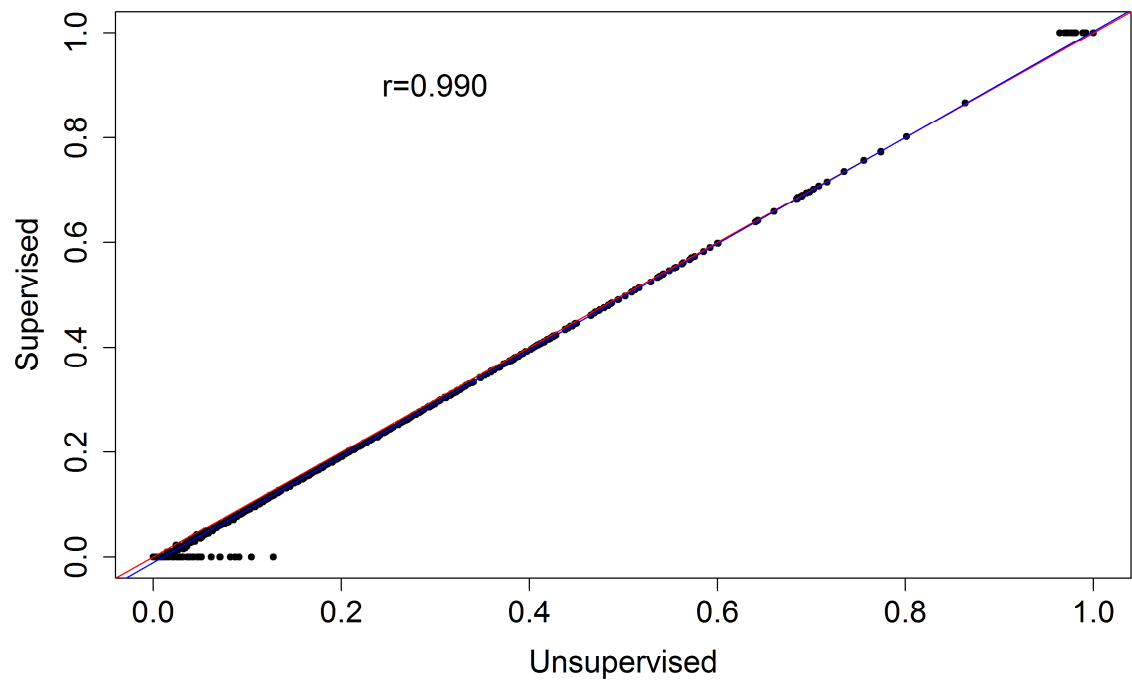

Supplement: Supplementary file 3 — Scatter plots of individual admixture levels estimated with supervised vs. unsupervised analyses. Blue line represents fitted linear regression line of supervised on unsupervised and red color represents the diagonal line when x = y. (PDF 86 kb) [file 12863_2017_526_MOESM3_ESM.pdf]

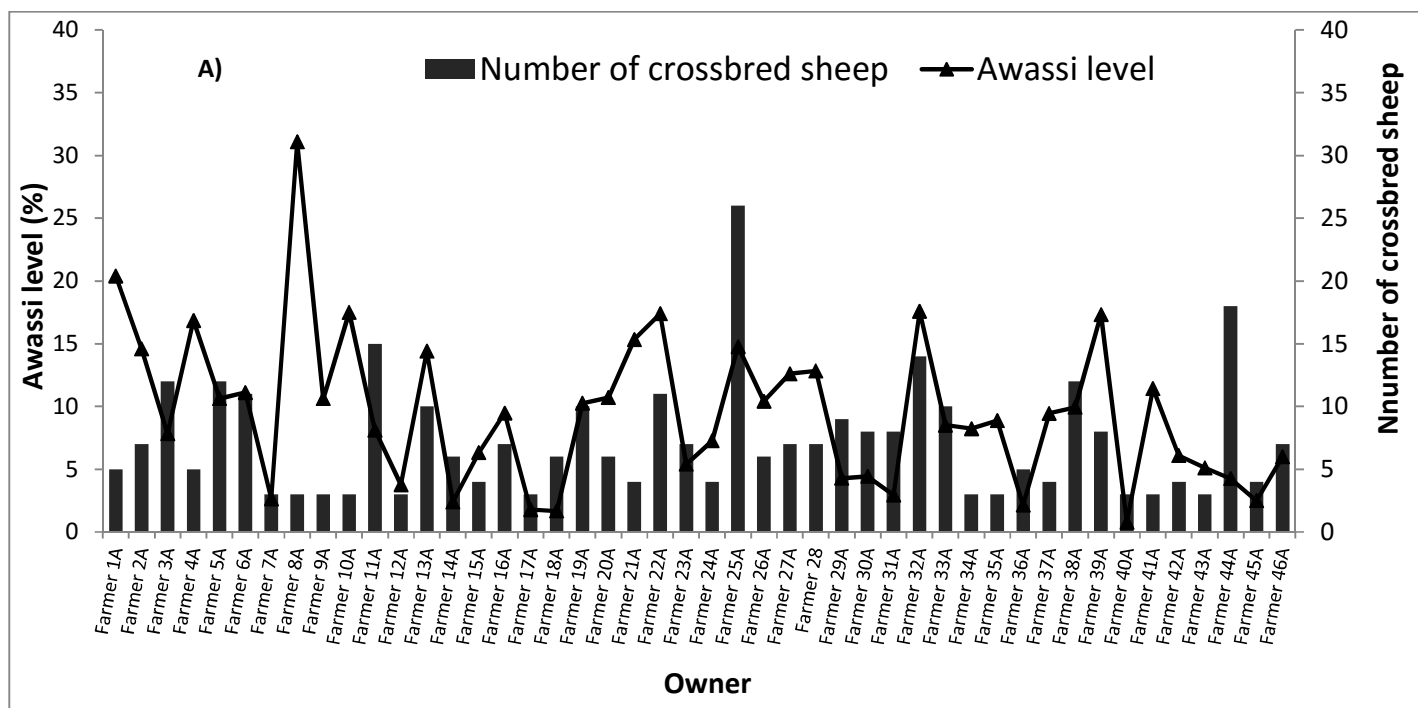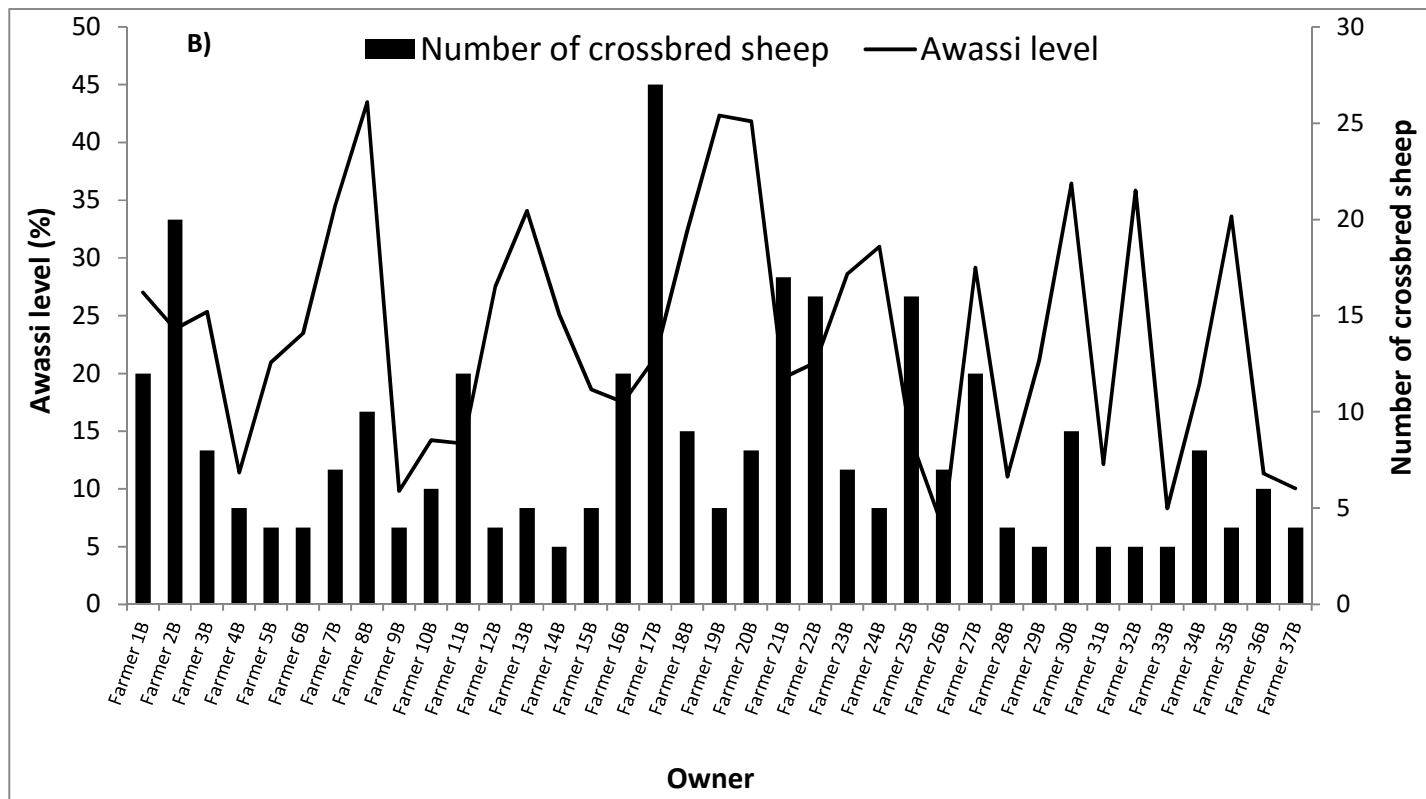

Supplement: Supplementary file 8 — Mean Awassi level and total number of crossbred sheep produced by farmers in Negasi-Amba (A) and Chiro (B) villages. Bar plots are indicated the total number of crossbred sheep by an owner and line plots showed the average level of Awassi (%). (PDF 234 kb) [file 12863_2017_526_MOESM8_ESM.pdf]
