# Supplementary material for: Identifying highly informative genetic markers for quantification of ancestry proportions in crossbred sheep populations: implications for choosing optimum levels of admixture
Source: BMC Genet. 2017 Aug 24;18:80. doi: 10.1186/s12863-017-0526-2 (PMC5571632; doi:10.1186/s12863-017-0526-2)
Supplement: Supplementary file 4 — Least square means ± standard errors of lamb eight months weight for crossbred population by location, different Awassi level groups and sex in Negasi-Amba and Chiro villages. (DOC 48 kb) [file 12863_2017_526_MOESM4_ESM.doc]

**Least square means (kg)±standard errors of lamb eight months weight for crossbred population by location, different Awassi level groups and sex in Negasi-Amba and Chiro villages**

| **Class** | **Negasi-Amba** | |  | **Chiro** | |  | **P value for location effect** | |
| --- | --- | --- | --- | --- | --- | --- | --- | --- |
|  | **N*^a^*** | **LSM*^b^*±SE*^c^*** |  | **N*^a^*** | **LSM*^b^*±SE*^c^*** |  |  |  |
| Overall | 179 | 16.3±0.35 |  | 149 | 20.4±0.36 |  | <0.0001 |  |
| CV*^d^* (%) |  | 21.1 |  |  | 28.4 |  |  |  |
| Range |  | 9.6-25.9 |  |  | 11.2-36.4 |  |  |  |
| Awassi level | * | |  | *** | |  |  |  |
| 0 | 62 | 15.2±0.42^a^ |  | 9 | 15.1±1.70 ^a^ |  |  |  |
| 0 to <12.5% | 63 | 16.8±0.41^b^ |  | 22 | 17.8±1.08^a^ |  |  |  |
| 12.5 to <25% | 35 | 17.1±0.55^b^ |  | 46 | 19.1±0.75^b^ |  |  |  |
| 25 to <37.5% | 4 | 17.0±1.65^b,a^ |  | 30 | 21.7±0.93^c^ |  |  |  |
| 37.5 to <50% | - | - |  | 18 | 24.8±1.20^d^ |  |  |  |
| ≥50% | - | - |  | 15 | 24.0±1.31^d^ |  |  |  |
| Sex | Ns | |  | * | |  |  |  |
| Male | 86 | 16.9±0.55 |  | 66 | 21.6±0.66 |  |  |  |
| Female | 78 | 16.2±0.50 |  | 74 | 19.3±0.64 |  |  |  |

*^a^*N: number of observations, *^b^*LSM: least square means (kg), *^c^*SE=standard error, *^d^*CV: coefficient of variation. *** Significant at *P*=0.001, *significant at *P=*0.05, ns=non-significant at *P=*0.05.
