# Supplementary material for: Identifying highly informative genetic markers for quantification of ancestry proportions in crossbred sheep populations: implications for choosing optimum levels of admixture
Source: BMC Genet. 2017 Aug 24;18:80. doi: 10.1186/s12863-017-0526-2 (PMC5571632; doi:10.1186/s12863-017-0526-2)
Supplement: Supplementary file 5 — Least square means ± standard errors of lambing interval, number of lambs weaned per ewe per year and body condition score for the effect of Awassi level groups and sex in each site. (DOC 45 kb) [file 12863_2017_526_MOESM5_ESM.doc]

**Least square means (kg)±s**tandard errors of lambing interval, number of lambs weaned per ewe per year and body condition score for the effect of Awassi level groups and sex in each site.

| **Location/Awassi level** | **LI*^a^*** | |  | **NLWEY*^e^*** | |  | **BC*^f^*** | |
| --- | --- | --- | --- | --- | --- | --- | --- | --- |
|  | **N*^b^*** | **LSM*^c^*±SE*^d^*** |  | **N** | **LSM*^c^*±SE*^d^*** |  | **N** | **LSM*^c^*±SE*^d^*** |
| **Negasi-Amba** | * | |  |  | ns | | ns | |
| 0 | 46 | 262±9.7^a^ |  | 34 | 1.25±0.06 |  | 28 | 2.5±0.12 |
| 0 to <12.5% | 28 | 290±12.5^a,b^ |  | 21 | 1.10±0.07 |  | 20 | 2.6±0.15 |
| 12.5 to <25% | 42 | 298±10.2^b^ |  | 33 | 1.20±0.06 |  | 24 | 2.4±0.13 |
| 25 to <37.5% | 10 | 303±20.9^a,b^ |  | 9 | 1.18±0.11 |  | 23 | 2.6±0.18 |
| 37.5 to <50% | - | - |  | - | - |  | - | - |
| **Chiro** | * | |  | ns | |  |  | ns |
| 0 | 27 | 283±13.7^a^ |  | 27 | 1.24±0.07 |  | 14 | 2.9±0.15 |
| 0 to <12.5% | 20 | 280±16.0^a^ |  | 20 | 1.26±0.09 |  | 12 | 2.4±0.16 |
| 12.5 to <25% | 38 | 297±11.6^a,b^ |  | 38 | 1.18±0.07 |  | 33 | 2.4±0.10 |
| 25 to <37.5% | 36 | 305±11.9^a,b^ |  | 36 | 1.19±0.07 |  | 32 | 2.4±0.10 |
| 37.5 to <50% | 18 | 334±16.8^b^ |  | 18 | 1.11±0.09 |  | 14 | 2.9±0.15 |

*^a^*LI: lambing interval, *^b^*N: number of observations, *^c^*LSM: least square means, *^d^*SE: standard error, *^e^*NLWEY: number of lambs weaned per ewe per year, *^f^*BC: body condition score. *significant at *P=*0.05, ns = non-significant at *P=*0.05.
