# Supplementary material for: Identifying highly informative genetic markers for quantification of ancestry proportions in crossbred sheep populations: implications for choosing optimum levels of admixture
Source: BMC Genet. 2017 Aug 24;18:80. doi: 10.1186/s12863-017-0526-2 (PMC5571632; doi:10.1186/s12863-017-0526-2)
Supplement: Supplementary file 6 — Least square means ± standard errors of eight months weight, body condition score (BC) and Awassi level for top ranked and poor performing lambs. (DOC 37 kb) [file 12863_2017_526_MOESM6_ESM.doc]

**Least square means (kg) ± standard errors** of eight months weight, body condition score (BC) and Awassi level for top ranked and poor performing lambs.

| **Performance level** | **Negasi-Amba** | | | | | **Chiro** | | | |
| --- | --- | --- | --- | --- | --- | --- | --- | --- | --- |
|  | **N** | **8 months weight (kg)** | **BC** | **Awassilevel (%)** |  | **N** | **8 months weight (kg)** | **BC** | **Awassilevel (%)** |
|  |  | *** | *** | ns |  |  | *** | *** | *** |
| Top | 22 | 22.7±0.37^a^ | 3.0±0.09^a^ | 10.1±1.50 |  | 19 | 30.6±0.84^a^ | 3.2± 0.11^a^ | 37.1± 3.51^a^ |
| Medium | 121 | 16.1±0.16^b^ | 2.9±0.04^a^ | 8.3± 0.63 |  | 98 | 19.8±0.35^b^ | 2.9± 0.05^b^ | 25.2± 0.54^b^ |
| Poor | 21 | 11.7±0.37^c^ | 2.6±0.09^b^ | 6.8± 1.47 |  | 25 | 13.9±0.78^c^ | 2.6± 0.10^c^ | 17.7± 0.00^c^ |
| **Overall** | **164** | **16.8±0.18** | **2.8±0.04** | **8.4± 0.73** |  | **142** | **20.7±0.38** | **2.9± 0.05** | **26.7± 1.62** |

*** Significant at *P*=0.001, *significant at *P=*0.05, ns=non-significant at *P=*0.05, N=number of observations, LSM=least square means, SE=standard error.
