# Supplementary material for: Identifying highly informative genetic markers for quantification of ancestry proportions in crossbred sheep populations: implications for choosing optimum levels of admixture
Source: BMC Genet. 2017 Aug 24;18:80. doi: 10.1186/s12863-017-0526-2 (PMC5571632; doi:10.1186/s12863-017-0526-2)
Supplement: Supplementary file 7 — Least square means ± standand error of Awassi level and reproductive performance for top, medium and worst performing ewes in Negasi-Amba and Chiro sites. (DOC 37 kb) [file 12863_2017_526_MOESM7_ESM.doc]

Least square means (kg) ± standard errors of Awassi level and reproductive performance for top, medium and worst performing ewes in Negasi-Amba and Chiro sites

| Performance level | Negasi-Amba | | | |  | Chiro | | | |
| --- | --- | --- | --- | --- | --- | --- | --- | --- | --- |
|  | N*^a^* | Awassi level (%) | LI*^b^*(days) | NLWEY*^c^* |  | N*^a^* | Awassi level (%) | LI*^b^*(days) | NLWEY*^c^* |
|  |  | ns | *** | *** |  |  | ns | *** | *** |
| Top | 24 | 9.0±1.97 | 227±10.1^a^ | 1.61±0.03^a^ |  | 20 | 15.9±3.1 | 216±11.6^a^ | 1.89±0.041^a^ |
| Medium | 56 | 10.6±1.36 | 283±6.7^b^ | 1.18±0.02^b^ |  | 96 | 20.3±1.4 | 301±5.5^b^ | 1.19±0.020^b^ |
| Poor | 24 | 12.8±1.97 | 356±10.1^c^ | 0.77±0.03^c^ |  | 22 | 21.3±2.9 | 367±11.6^c^ | 0.69±0.041^c^ |
| Overall | 104 | 10.8±1.03 | 289±5.2 | 1.18±0.015 |  | 132 | 19.2±1.5 | 295±5.78 | 1.26±0.021 |

*^a^*N=number of observations, *^b^*LI=lambing interval, *^c^*NLWEY=number of lambs weaned ewe^-1^ year^-1^,*** Significant at *P*=0.001, *significant at *P=*0.05, ns=non-significant at *P=*0.05,
